# Prognostic value of systemic inflammation response index in hepatoblastoma patients receiving preoperative neoadjuvant chemotherapy

**DOI:** 10.3389/fonc.2023.1276175

**Published:** 2023-10-13

**Authors:** Chen Zheng, Shiru Ye, Wei Liu, Mei Diao, Long Li

**Affiliations:** ^1^ Department of Pediatric Surgery, Capital Institute of Pediatrics, Beijing, China; ^2^ Graduate School of Peking Union Medical College, Chinese Academy of Medical Sciences, Beijing, China; ^3^ Research Unit of Minimally Invasive Pediatric Surgery on Diagnosis and Treatment, Chinese Academy of Medical Sciences, Beijing, China

**Keywords:** systemic inflammation response index, hepatoblastoma, neoadjuvant chemotherapy, prognosis, nomogram

## Abstract

**Introduction:**

Inflammation is closely associated with tumor development and patient prognosis. The objective of this study is to assess the prognostic value of the preoperative inflammatory indexes in pediatric hepatoblastoma patients who receive neoadjuvant chemotherapy.

**Methods:**

A retrospective analysis was performed on clinical and pathological data of 199 hepatoblastoma patients who underwent hepatectomy with preoperative neoadjuvant chemotherapy from January 2015 to June 2020. The receiver operating characteristic curve was used to evaluate the prognostic value of neutrophil-to-lymphocyte ratio (NLR), platelet-to-lymphocyte ratio (PLR), systemic immune-inflammation index (SII), and systemic inflammation response index (SIRI) in predicting OS and EFS. Patients were grouped based on optimal cutoff values of preoperative inflammatory indexes. Survival rates were calculated using the Kaplan-Meier method, and survival outcomes were compared between groups using the log-rank test. Univariate and multivariate Cox proportional hazards regression models were used to identify independent prognostic factors, and a nomogram was constructed using R software to predict the probability of OS.

**Results:**

The receiver operating characteristic curve showed prognostic value for OS, not EFS, in preoperative inflammatory indexes. Patients were categorized into low/high groups: SII ≤ 266.70/higher, NLR ≤ 1.24/higher, PLR ≤ 85.25/higher, and SIRI ≤ 0.72/higher. High NLR, PLR, SII, and SIRI groups had significantly lower 5-year OS than their low counterparts (all *p*-value < 0.05). The Cox analysis identified four independent prognostic factors: SIRI (HR=2.997, 95% CI: 1.119-8.031), microvascular invasion (HR=2.556, 95% CI: 1.14-5.73), the post-treatment extent of disease (POSTTEXT) staging (IV *vs.* I: HR=244.204, 95% CI:11.306-5274.556), and alpha-fetoprotein (>100 ng/ml: HR=0.11, 95% CI: 0.032-0.381) for hepatoblastoma patients with neoadjuvant chemotherapy. High SIRI group had more patients with adverse NLR, SII, and POSTTEXT III (all *p*-value < 0.05). Independent prognostic factors led to an OS nomogram with a concordance index of 0.85 (95% CI: 0.78-0.91, *p*-value = 1.43e-27) and the calibration curve showed a good fit between the prediction curve and the true curve.

**Conclusions:**

SIRI is an independent prognostic factor of hepatoblastoma patients receiving neoadjuvant chemotherapy. The OS nomogram based on SIRI, POSTTEXT staging, MiVI, and AFP can be used to assess the prognosis of those patients.

## 1 Introduction

Hepatoblastoma (HB) is the most common primary liver malignant tumor in children, ranking as the third most prevalent pediatric abdominal malignancy after Wilms’ tumor and neuroblastoma. It accounts for approximately 1% of all pediatric cancers, and its incidence is on the rise (1–3). HB typically occurs in children under 3 years old, with a male-to-female ratio of approximately 1.5:1. The disease is often associated with premature birth, low birth weight, heredity and chromosome abnormalities, although the specific etiology remains unclear (4, 5). HB is sensitive to chemotherapy, and preoperative neoadjuvant chemotherapy (NACT) plays a crucial role in down-grading the disease staging, improving the rate of complete tumor resection during surgery, and lowering tumor recurrence rates (6). Therefore, preoperative NACT has been widely adopted in clinical practice and gained significant recognition. The combination of surgery and NACT has significantly improved postoperative survival in high-risk children with HB (1). Currently, the pretreatment extent of disease (PRETEXT)/the post-treatment extent of disease (POSTTEXT) staging based on imaging is widely used in prognosis prediction and treatment decision-making of HB patients (7–9). The PRETEXT staging is determined by imaging at diagnosis and the POSTTEXT staging is determined after neoadjuvant chemotherapy. When feasible and safe, the Children’s Oncology Group (COG) strategy recommends upfront resection at diagnosis (mainly PRETEXT I and PRETEXT II) to minimize overall chemotherapy exposure (10–12), and preoperative NACT or liver transplantation for those patients with unresectable hepatoblastoma. However, in clinical practice, the prognosis of patients who do not clearly respond to neoadjuvant therapy may vary considerably despite having the same POSTTEXT staging. This disparity can be attributed to several underlying biological factors. Consequently, there exists an imperative clinical demand for the establishment of biomarkers capable of identifying HB patients with a bleak prognosis. Recently, several immunological and histological biomarkers have been identified for assessing the prognosis of HB patients (13–15). Nevertheless, these biomarkers frequently rely on primary tumor samples, entail technical complexity, and incur substantial costs. Therefore, it is of paramount importance to explore a convenient, cost-effective, and efficient preoperative biomarker that can assist surgeons in the identification of HB patients at a higher risk. Furthermore, this endeavor could facilitate the prognostic assessment of HB patients predicted to have poor survival outcomes, allowing the development of personalized follow-up plans to maximize benefit for these individuals.

The systemic inflammation response index (SIRI) is a hematological index established based on the counts of neutrophils, monocytes, and lymphocytes in peripheral blood to evaluate the inflammatory state of the body. It serves as a useful predictor of poor prognosis in the treatment of malignant tumors (16). Previous research has indicated that the inflammatory process can activate oncogenic signaling pathways, promoting tumor growth and metastasis, thereby leading to poor outcomes (17–19). SIRI effectively reflects the body’s inflammatory response and immune status. An elevated SIRI indicates increased neutrophil and monocyte counts, as well as decreased lymphocyte counts, which are conducive to tumor development and metastasis (20–23). Numerous studies in the field of adult oncology have confirmed the association between elevated preoperative SIRI and adverse clinical outcomes (24–27).

Therefore, the objective of this study is to analyze the correlation between preoperative SIRI and the prognosis of HB patients who received NACT. Additionally, we aim to evaluate the clinical value of SIRI as a preoperative biomarker for predicting the prognosis of these patients and to compare it with other common inflammatory indexes.

## 2 Materials and methods

### 2.1 Patients

This study retrospectively analyzed pediatric HB patients who underwent hepatectomy at our Institution from January 2015 to June 1, 2020. Ethical approval was obtained from the Ethics Committee of the Capital Institute of Pediatrics, and written informed consent was obtained from the parents before surgery.

Inclusion criteria were as follows:

1. Pathologically confirmed hepatoblastoma.

2. Completion of preoperative neoadjuvant chemotherapy including either the PLADO (cisplatin and doxorubicin) or C5 V (cisplatin, 5-flourouracil, and vincristine) regimen.

3. Primary radical resection of the tumor.

4. Age less than 18 years.

Exclusion criteria were as follows:

1. Coexisting malignancies in other systems.

2. Perioperative death (≤60 days).

3. Incomplete clinical and follow-up data.

4. Previous hepatectomy for other diseases.

### 2.2 Clinical and pathological data

Clinical information was collected from electronic medical records, including age, gender, initial alpha-fetoprotein (AFP) levels and preoperative imaging data. The preoperative imaging data included the PRETEXT/POSTTEXT staging, tumor location, multifocality (F+) including two or more tumor nodules separated by normal hepatic parenchyma, macrovascular involvement (MaVI) including either hepatic venous involvement (V+) or portal venous involvement (P+), extrahepatic intra-abdominal disease (E+), tumour rupture or intraperitoneal haemorrhage(H+) and distant metastasis. Based on the International Pediatric Liver Tumor Strategy Group (SIOPEL) guidelines, the POSTTEXT staging was used for those HB patients who received preoperative NACT (28, 29). Pathological data included tumor histological types and microvascular invasion (MiVI), which was defined as pathologic vascular invasion noted microscopically by the examining pathologist (30). According to the Chinese Guidelines for the Diagnosis and Treatment of Pediatric Hepatoblastoma (2019 edition) and the prognostic characteristics of different pathological types (31, 32), the HB histological types were classified into three categories: small cell undifferentiated (SCUD) HB, other epithelial HB excluding SCUD, and mixed epithelial-mesenchymal (MEM) HB. all tumor samples were reviewed in detail by at least two pathologists, and by three in case of uncertainty.

### 2.3 Preoperative inflammatory indexes

The preoperative inflammatory indexes (Collected within 1 week before surgery) included the Neutrophil-to-Lymphocyte Ratio (NLR), Platelet-to-Lymphocyte Ratio (PLR), Systemic Immune-Inflammation Index (SII), and Systemic Inflammation Response Index (SIRI). NLR was calculated as neutrophil counts/lymphocyte counts, PLR as platelet counts/lymphocyte counts, SII as platelet counts × neutrophil counts/lymphocyte counts, and SIRI as monocyte counts × neutrophil counts/lymphocyte counts.

### 2.4 Follow-up

Patients were followed up regularly through outpatient visits and telephone contacts. Within the first year after surgery, liver function, AFP levels, and upper abdominal ultrasound, computed tomography (CT) or magnetic resonance imaging (MRI) examinations were performed every 3 months. Subsequently, follow-up occurred every 3 to 6 months after the first year. The follow-up period ended on July 1, 2023. The primary endpoints were overall survival (OS), defined as the time from the first hepatectomy to death, and event-free survival (EFS), defined as the first hepatectomy to the diagnosis of relapse, metastasis, or death.

### 2.5 Statistical analysis

Statistical analyses were performed using SPSS (version 26, Chicago, Illinois) and R software (version 4.3.1, http://www.r-project.org). Continuous data are reported as mean ± standard deviation or median (interquartile range [IQR]). Categorical variables were presented as frequencies (n) and percentages (%). Student’s t-test was used for comparison of normally distributed continuous variables and Wilcoxon rank sum test for non-normally distributed continuous variables. The Chi-square test was used to compare categorical variables. The Mann-Whitney U test was used to compare group-ranked data. Receiver operating characteristic (ROC) curve was used to calculate the area under the curve (AUC) of preoperative NLR, PLR, SIRI and SII for OS and EFS in HB patients. A minimum AUC > 0.7 was considered clinically significant (33).The optimal cut-off values for inflammatory indexes were determined using the Youden index to stratify patients for further analysis (34, 35). The Kaplan-Meier (KM) curve was used to calculate survival rates, and the log-rank test was used to compare survival outcomes between groups. The Cox proportional hazards regression model was used for univariate and multivariate analysis of prognostic factors. Based on the independent prognostic factors identified by the Cox proportional hazards regression model, the “rms” package in R was used to draw calibration plots for the prediction model and to calculate the concordance index (C-index) to determine the discrimination of the prediction model. Consistency analysis was performed by plotting calibration curves. The clinical utility of the nomogram was evaluated by decision curve analysis (DCA) using the “devtools” package. In all of the above analyses, *p*-value < 0.05 was considered statistically significant.

## 3 Results

### 3.1 Baseline data

A total of 199 patients were enrolled in this study, including 125 males (62.8%) and 74 females (37.2%), with a median age of 20.0 months (range, 0.1-124.0 months) and an IQR of 21.0 months. According to SIOPEL criteria, before NACT, 5 patients were PRETEXT I (2.5%), 61 patients were PRETEXT II (30.6%), 102 patients were PRETEXT III (51.3%), and 31 patients were PRETEXT IV (15.6%). Prior to surgery, 19 patients were classified as POSTTEXT I (9.5%), 147 as POSTTEXT II (73.9%), 31 as POSTTEXT III (15.6%), and 2 as POSTTEXT IV (1%). Intrahepatic multifocality (F+) were identified in 38 patients (19.1%), extrahepatic intra-abdominal disease (E+) in 11 patients (5.5%) and tumour rupture or intraperitoneal haemorrhage (H+) in 8 patients (4.0%). 32 patients had distant metastasis (16.1%), and 58 cases of macrovascular involvement (29.1%). The results of the pathological analysis showed that there were 5 cases of SCUD HB (2.5%), 88 cases of epithelial HB other than SCUD (44.2%), mixed epithelial-mesenchymal HB in 106 cases (53.3%). Microscopic microvascular invasion was positive in 94 cases (47.2%). The median follow-up time for all patients was 60.1 months (time range 2-102 months), with an IQR of 42.9 months. During the follow-up period, 62 patients (31.1%) had tumor recurrence, of which 41 patients (20.6%) died as a result of tumor progression. The 1-, 3-, and 5-year OS rates were 81%, 80%, and 79%, respectively. The EFS rates at 1, 3, 5 years were 73%, 69% and 69%, respectively.

### 3.2 ROC analysis of inflammatory indexes

**Figure 1:**
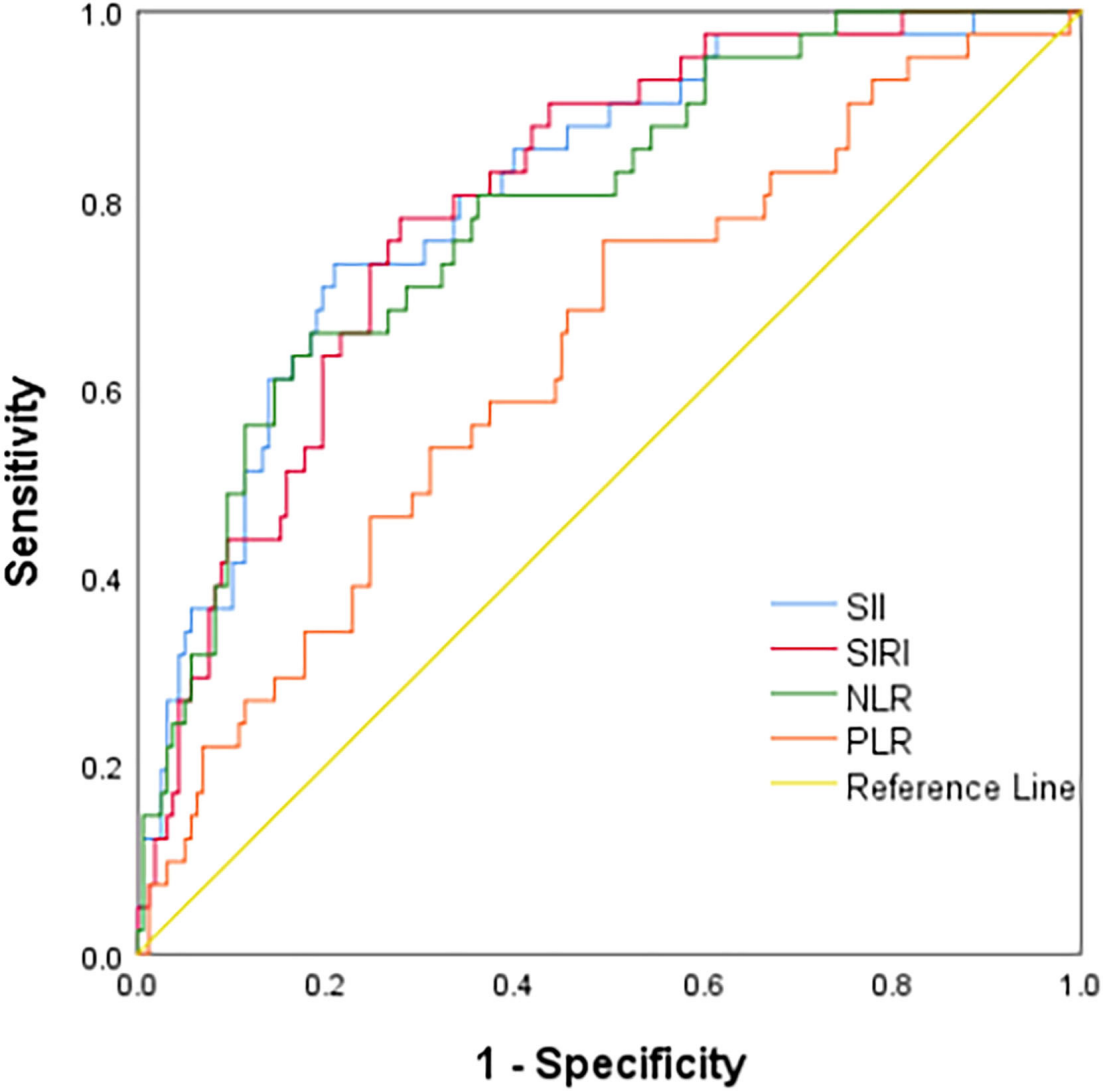
ROC curve of Inflammatory indexes for the overall survival.

**Figure 2:**
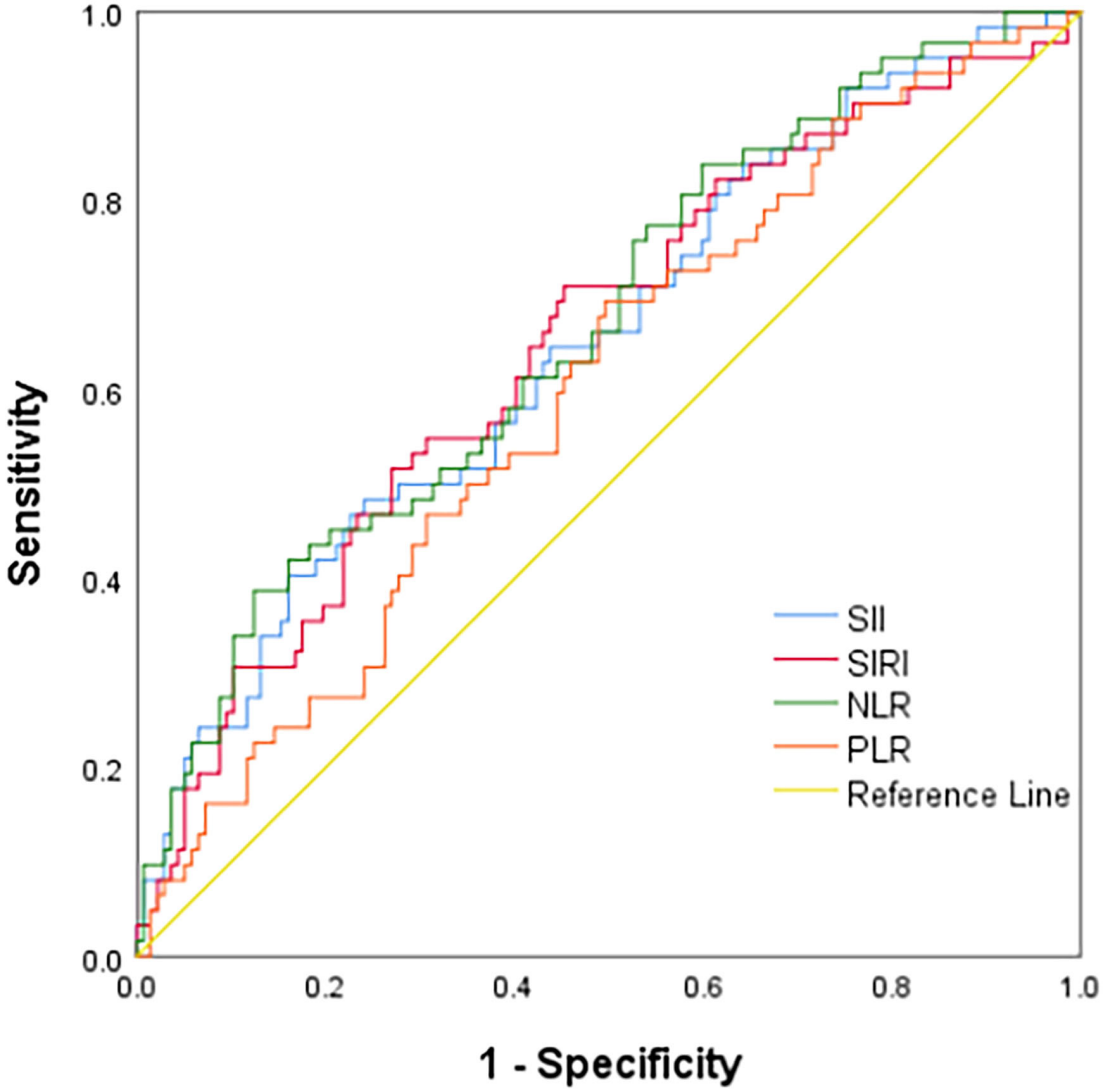
ROC curve of Inflammatory indexes for the event-free survival.

ROC curve analysis showed that the AUCs for predicting postoperative OS in HB patients were 78.9% (95% CI: 71.3%-86.5%) for NLR, 63.7% (95% CI: 54.3%-73.2%) for PLR, 80.6% (95% CI: 73.4%-87.9%) for SII, and 79.8% (95% CI: 72.9%-86.8%) for SIRI. The optimal cutoff values (**Table 1**) were 1.24 for NLR, 85.25 for PLR, 266.70 for SII, and 0.72 for SIRI. The AUCs for SIRI, SII, and NLR were all greater than 0.7, indicating that these preoperative inflammatory indexes had prognostic value for OS (**Figure 1**). The AUCs for NLR, PLR, SII, and SIRI for predicting postoperative EFS were 66.5% (95% CI: 58.4%-74.6%), 59.9% (95% CI: 51.6%-68.2%), 64.9% (95% CI: 56.7%-73.2%), and 64.9% (95% CI: 56.6%-73.2%), respectively, all less than 0.7 (**Table 2**), indicating poor prognostic performance for EFS (**Figure 2**).

**Table 1:** ROC Analysis of Inflammatory indexes for the overall survival.

Variable	AUC	sensitivity	specificity	cut-off value	95% Confidence Interval	
					Lower Bound	Upper Bound
SII	0.806	0.732	0.790	266.700	0.734	0.879
NLR	0.789	0.659	0.820	1.240	0.713	0.865
PLR	0.637	0.756	0.510	85.250	0.543	0.732
SIRI	0.798	0.780	0.720	0.720	0.729	0.868

ROC, receiver operating characteristic; AUC, area under the curve.

**Table 2:** ROC Analysis of Inflammatory indexes for the event-free survival.

Variable	AUC	sensitivity	specificity	cut-off value	95% Confidence Interval	
					Lower Bound	Upper Bound
SII	0.649	0.484	0.759	266.700	0.567	0.732
NLR	0.665	0.387	0.876	1.511	0.584	0.746
PLR	0.599	0.694	0.504	84.207	0.516	0.682
SIRI	0.649	0.710	0.547	0.444	0.566	0.732

ROC, Receiver operating characteristic; AUC, area under the curve.

### 3.3 Prognostic factors for overall survival

**Figure 3:**
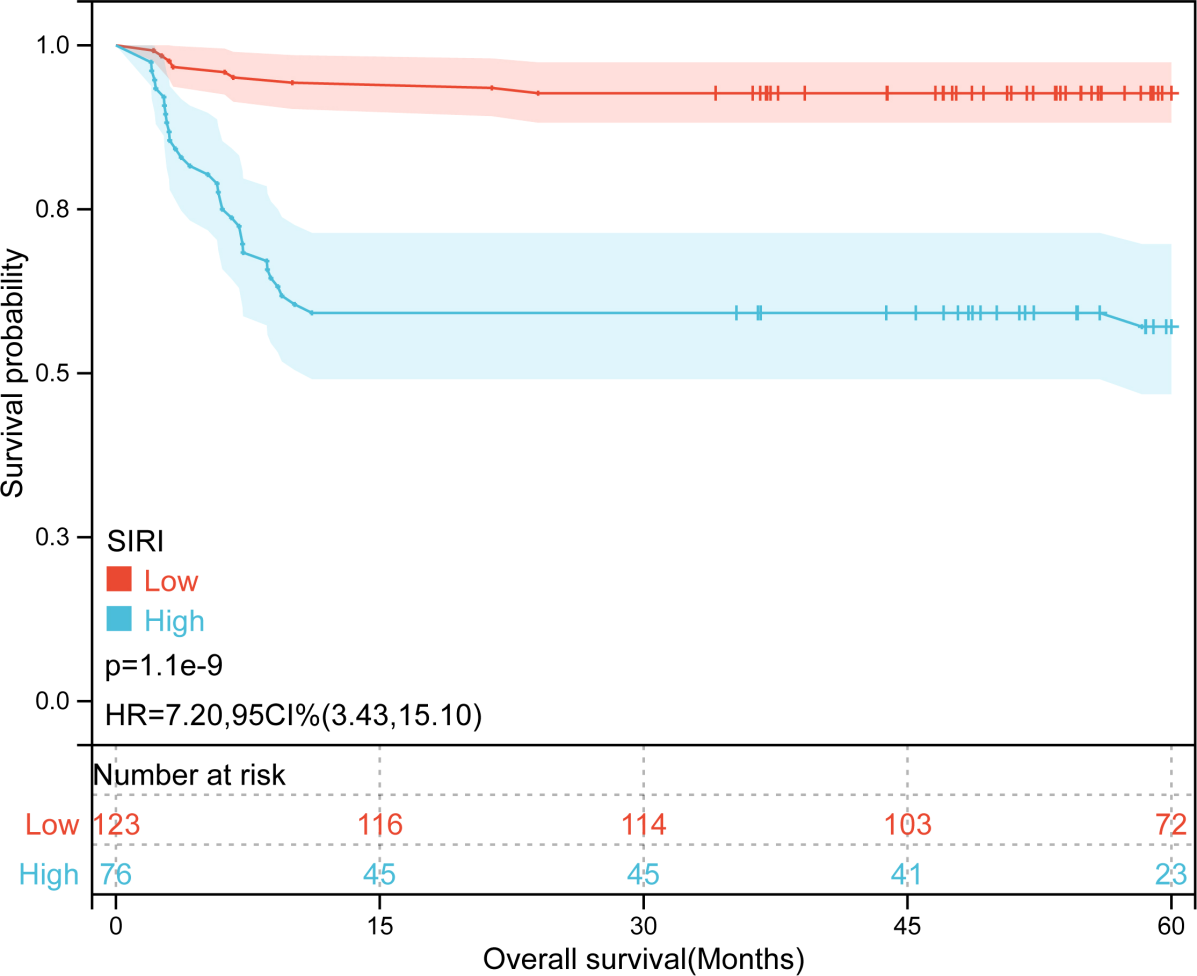
Kaplan-Meier curves for overall survival probability according to SIRI level.

**Figure 4:**
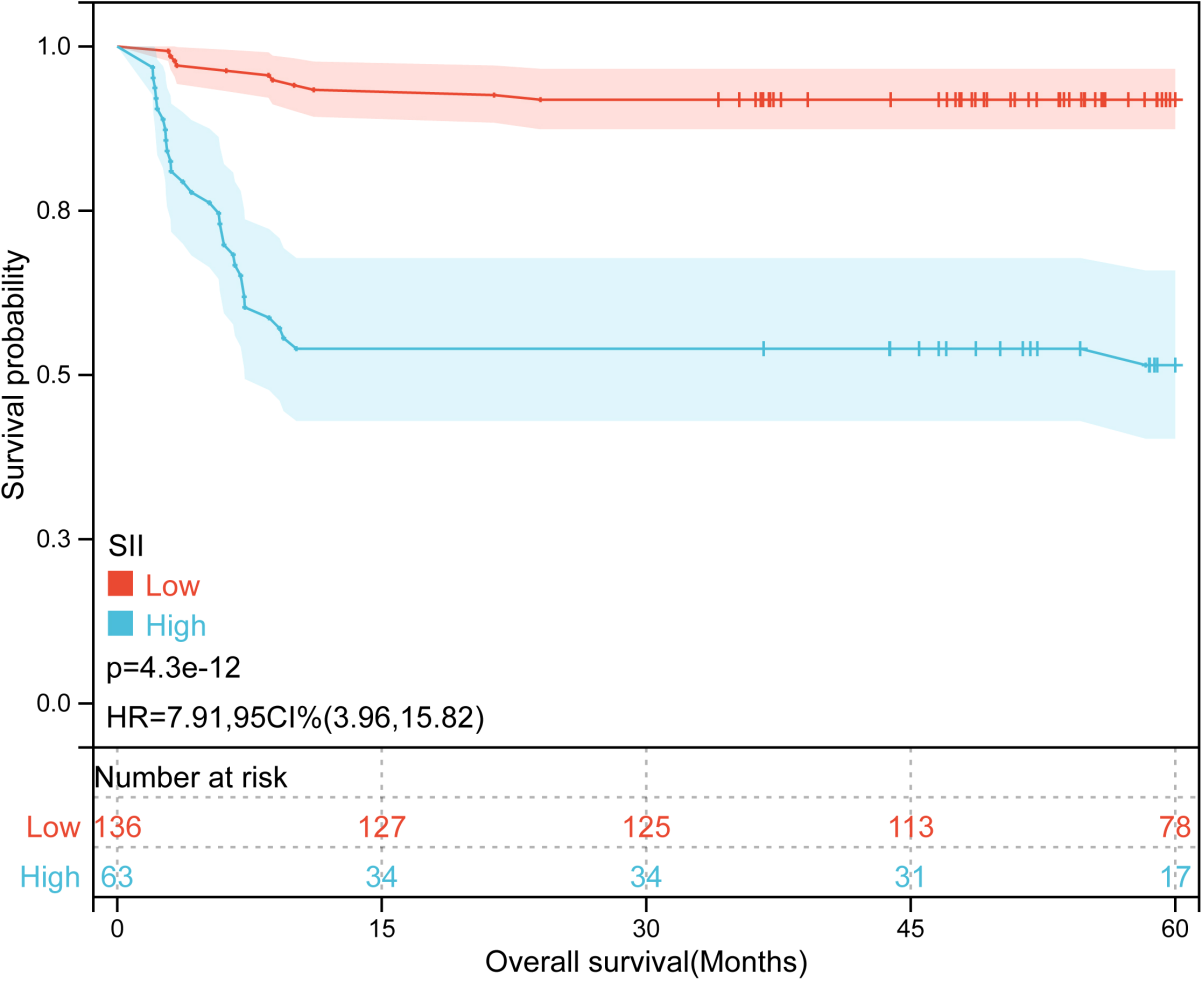
Kaplan-Meier curves for overall survival probability according to SII level.

**Figure 5:**
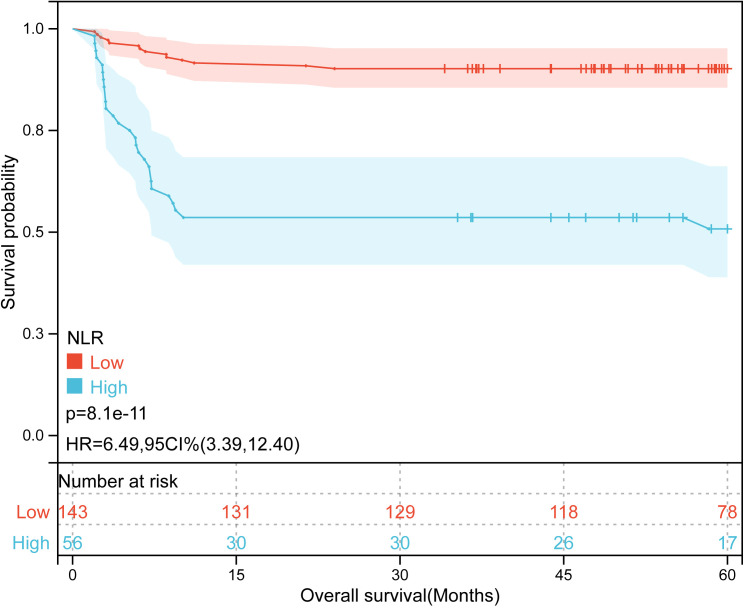
Kaplan-Meier curves for overall survival probability according to NLR level.

**Figure 6:**
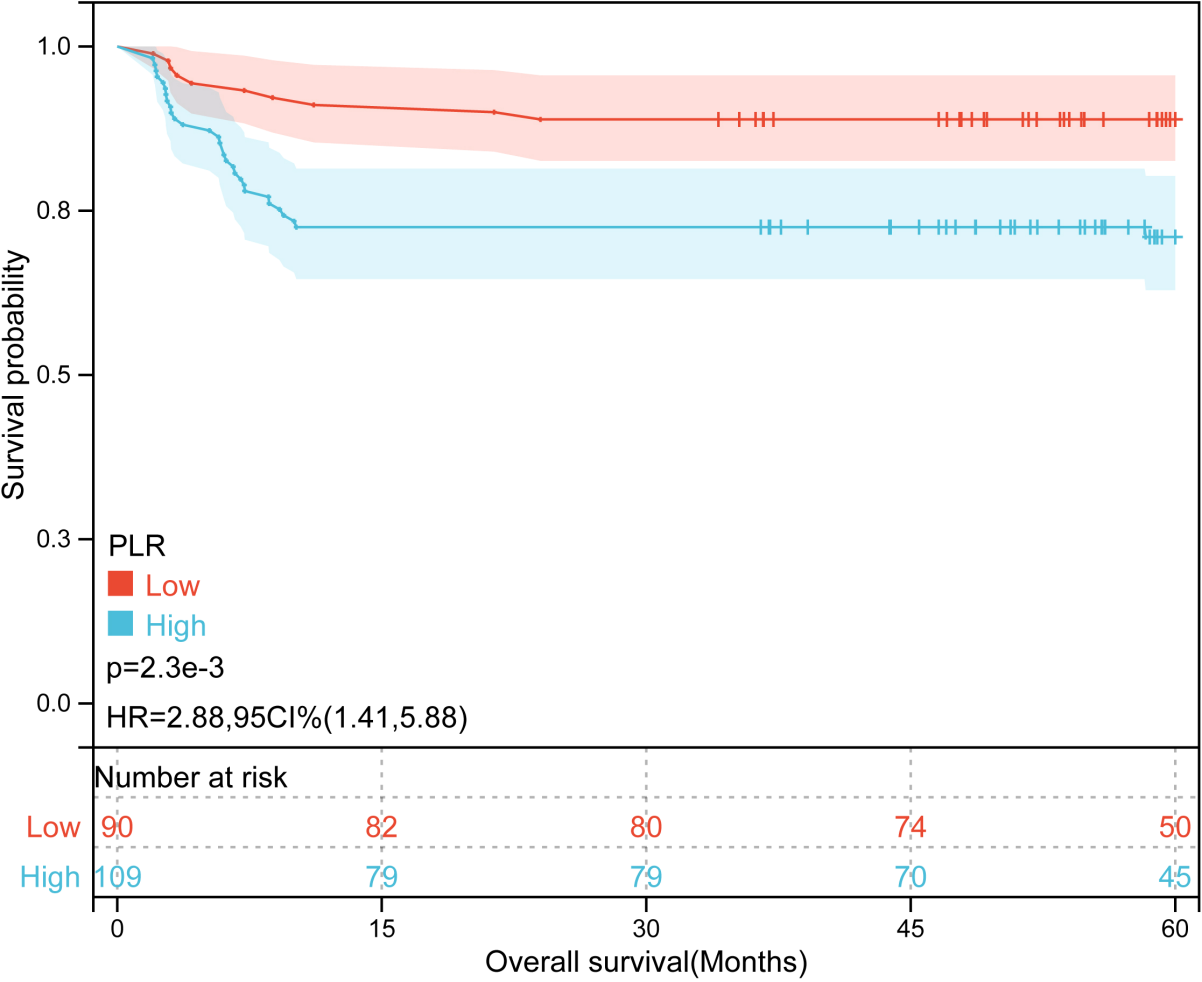
Kaplan-Meier curves for overall survival probability according to PLR level.

Kaplan-Meier plots (**Figures 3**–**6**) demonstrated that the 5-year OS of the high SII group, high NLR group, high PLR group, and high SIRI group was lower than that of the low SII group, low NLR group, low PLR group, and low SIRI group (all *p*-value < 0.05). Univariate Cox regression analysis revealed that NLR, PLR, SII, SIRI, POSTTEXT staging, AFP, metastasis, microvascular invasion, macrovascular involvement, and multifocality were associated with OS (all *p*-value < 0.05, **Table 2**). Multivariate Cox regression analysis indicated that SIRI (HR=2.997, 95% CI: 1.119-8.031), MiVI (HR=2.556, 95% CI: 1.14-5.73), POSTTEXT staging (IV *vs.* I: HR=244.204, 95% CI:11.306-5274.556), and AFP (>100 ng/ml: HR=0.11, 95% CI: 0.032-0.381) were independent prognostic factors for OS (all *p*-value < 0.05, **Table 3**). Among the preoperative inflammatory indexes, only SIRI was an independent prognostic factor for OS.

**Table 3:** Univariate and multivariate Cox analyses for the prediction of OS.

Cox for OS Variables	Univariate analysis		Multivariate analysis	
	HR (95% CI)	*P* value	HR (95% CI)	*P* value
POSTTEXT				
POSTTEXT I	reference			
POSTTEXT II	3.009(0.406-22.327)	0.281	3.314(0.360-30.463)	0.29
POSTTEXT III	12.434(1.647-93.900)	**0.015**	6.130(0.568-66.187)	0.135
POSTTEXT IV	500.473(31.234-8019.300)	**< 0.001**	244.204(11.306-5274.556)	**< 0.001**
Metastasis	3.167(1.659-6.048)	**< 0.001**		
Multifocality	2.754 (1.458-5.204)	**0.002**		
MiVI	3.525 (1.766-7.037)	**< 0.001**	2.556(1.14-5.73)	**0.023**
AFP	0.187(0.086-0.407)	**< 0.001**	0.11(0.032-0.381)	**< 0.001**
Histology				
Epithelial (exclude SCUD)	reference			
MEM	0.581(0.303-1.113)	0.102		
SCUD	6.488(2.210-19.049)	**< 0.001**		
MaVI	1.868(1.004-3.479)	**0.049**		
E+	1.553(0.479-5.032)	0.463		
SII	7.898 (3.95-15.793)	**< 0.001**		
NLR	6.476 (3.388-12.377)	**< 0.001**		
PLR	2.88 (1.412-5.877)	**0.004**		
SIRI	1.117(1.071-1.165)	**< 0.001**	2.997(1.119-8.031)	**0.029**
Gender	1.012 (0.536-1.911)	0.971		
Age	1.302 (0.622-2.728)	0.484		
H+	1.256 (0.303-5.201)	0.753		

AFP, alpha-fetoprotein; CI, confidence interval; HR, hazard ratio; MaVI, macrovascular involvement including either hepatic venous involvement or portal venous involvement; E+, extrahepatic intra-abdominal disease; MEM, mixed epithelial and mesenchymal; MiVI, microvascular invasion; NLR, neutrophil-to-lymphocyte ratio; OS, overall survival; POSTTEXT, posttreatment extent of disease; PLR, platelet-to-lymphocyte ratio; SCUD, small cell undifferentiated; SII, systemic immune-inflammation index; SIRI, systemic immune-inflammation index; H+, tumour rupture or intraperitoneal haemorrhage. The P values < 0.05 were bolded.

### 3.4 Relationship between clinicopathological features of different SIRI groups

According to the optimal cutoff value for predicting OS in HB patients using SIRI, patients were divided into the low SIRI group (SIRI ≤0.72) and the high SIRI group (SIRI > 0.72). The proportions of patients with NLR > 1.24, SII > 266.70, and POSTTEXT III were higher in the high SIRI group than in the low SII group (all *p*-value < 0.05, **Table 4**).

**Table 4:** Association of the patients’characteristics with the SIRI .

Characteristics	High SIRI (N=76)	Low SIRI (N=123)	Total (N=199)	P value
Age (months)	21.00 [0.10,124.00]	20.00 [3.00,120.00]	20.00 [0.10,124.00]	0.28
Gender				0.71
Female	30 (39.47%)	44 (35.78%)	74 (37.2%)	
Male	46 (60.52%)	79 (64.2%)	125 (62.8%)	
POSTTEXT				0.03
POSTTEXT I	8 (10.5%)	11 (8.9%)	19 (9.5%)	
POSTTEXT II	48 (63.2%)	99 (80.5%)	147 (73.9%)	
POSTTEXT III	19 (25%)	12 (9.8%)	31 (15.6%)	
POSTTEXT IV	1 (1.3%)	1 (0.8%)	2 (1.0%)	
Metastasis				0.61
No	62 (81.6%)	105 (85.4%)	167 (83.9%)	
Yes	14 (18.4%)	18 (14.6%)	32 (16.1%)	
Multifocality				0.27
No	58 (76.3%)	103 (83.7%)	161 (80.9%)	
Yes	18 (23.7%)	20 (16.3%)	38 (19.1%)	
AFP (ng/ml)				0.13
≤100	8 (10.5%)	5 (4.1%)	13 (6.5%)	
>100	68 (89.5%)	118 (95.9%)	186 (93.5%)	
Histology				0.12
Epithelial (exclude SCUD)	35 (46.1%)	53 (43.1%)	88 (44.2%)	
MEM	37 (48.7%)	69 (56.1%)	106 (53.3%)	
SCUD	4 (5.3%)	1 (0.8%)	5 (2.5%)	
MaVI				0.45
No	51 (67.1%)	90 (73.2%)	141 (70.8%)	
Yes	25 (32.9%)	33 (26.8%)	58 (29.2%)	
MiVI				0.1
No	34 (44.7%)	71 (57.7%)	105 (52.8%)	
Yes	42 (55.3%)	52 (42.3%)	94 (47.2%)	
E+				0.34
No	70 (92.1%)	118 (95.9%)	188 (94.5%)	
Yes	6 (7.9%)	5 (4.1%)	11 (5.5%)	
SII				<0.001
Low	27 (35.5%)	109 (88.6%)	136 (68.3%)	
High	49 (64.5%)	14 (11.4%)	63 (31.7%)	
NLR				<0.001
Low	27 (35.5%)	116 (94.3%)	143 (71.9%)	
High	49 (65.5%)	7 (5.7%)	56 (28.1%)	
PLR				0.58
Low	32 (42.1%)	58 (47.2%)	90 (45.2%)	
High	44 (57.9%)	65 (52.8%)	109 (54.8%)	
H+				0.967
No	73 (96.1%)	118 (95.9%)	191 (96.0%)	
Yes	3 (3.9%)	5 (4.1%)	8 (4.0%)	

AFP, alpha-fetoprotein; MaVI, macrovascular involvement including either hepatic venous involvement or portal venous involvement, MEM, mixed epithelial and mesenchymal; MiVI, microvascular invasion; E+, extrahepatic intra-abdominal disease; NLR, neutrophil-to-lymphocyte ratio; POSTTEXT, posttreatment extent of disease; PLR, platelet-to-lymphocyte ratio; SCUD, small cell undifferentiated; SII, systemic immune-inflammation index; SIRI, systemic immune-inflammation index; H+, tumour rupture or intraperitoneal haemorrhage.

### 3.5 Nomogram predictive model for overall survival

**Figure 7:**
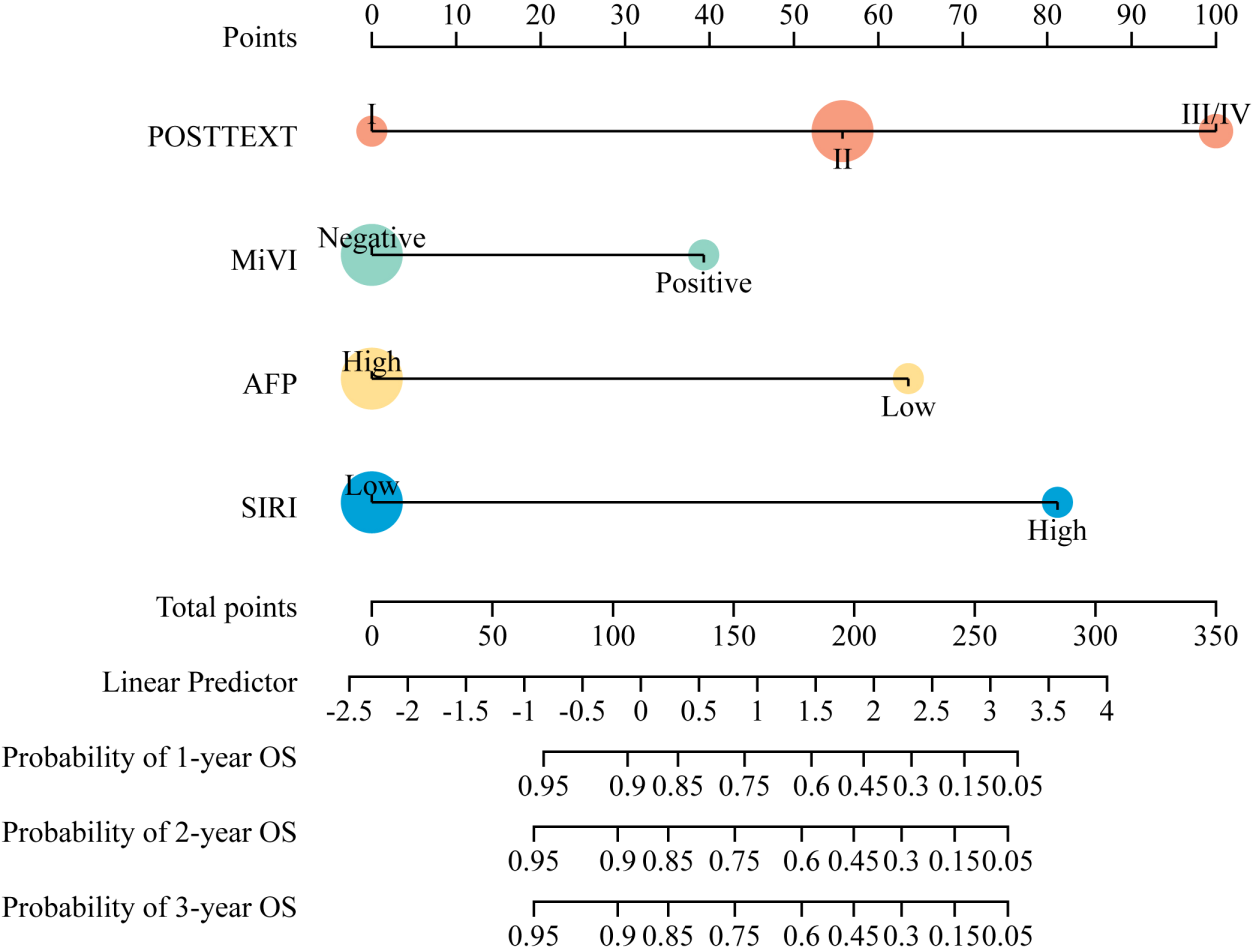
Nomogram for the probability of OS in HB patients receiving preoperative NACT.

**Figure 8:**
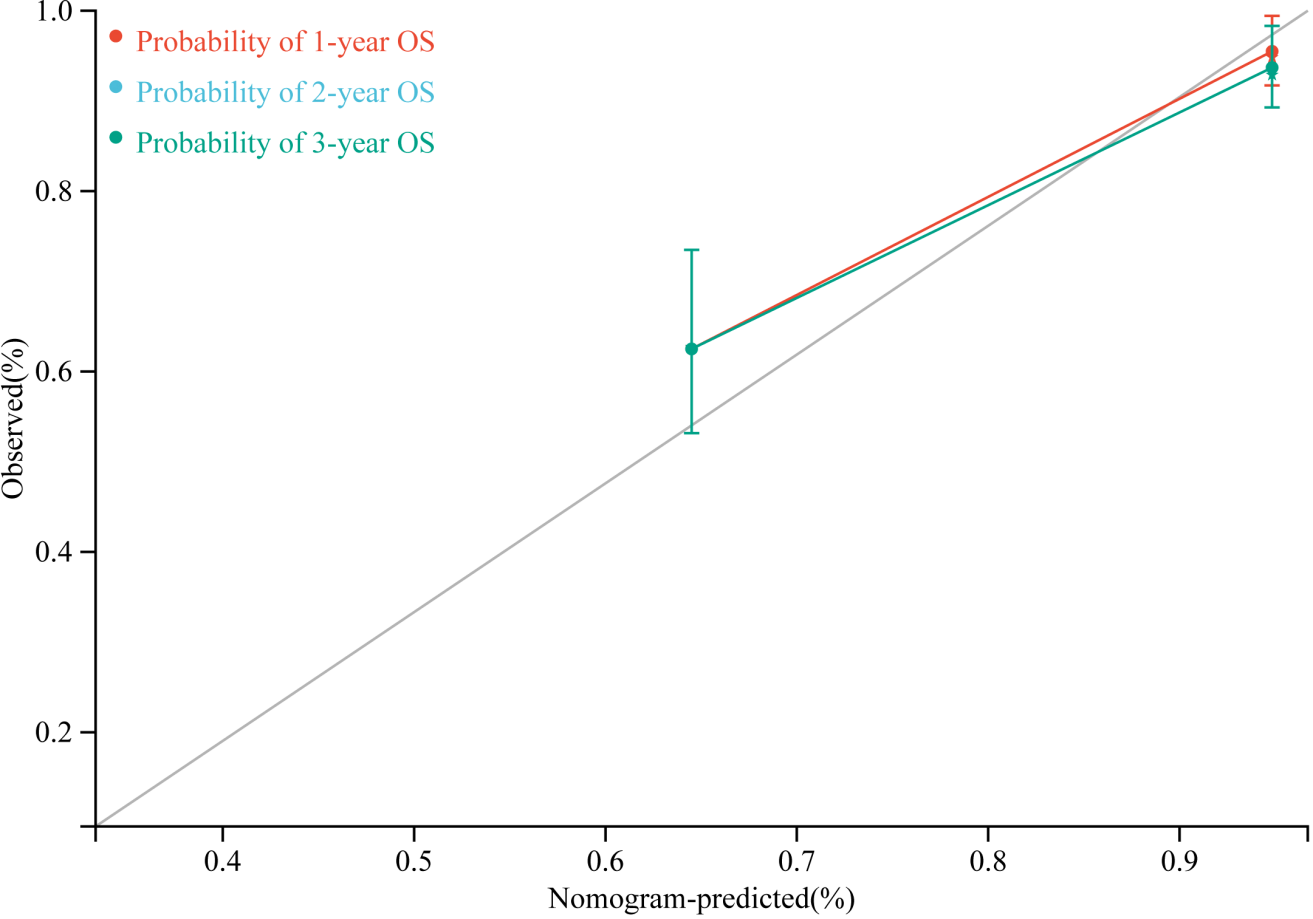
Predictive calibration curves for the nomogram.

**Figure 9:**
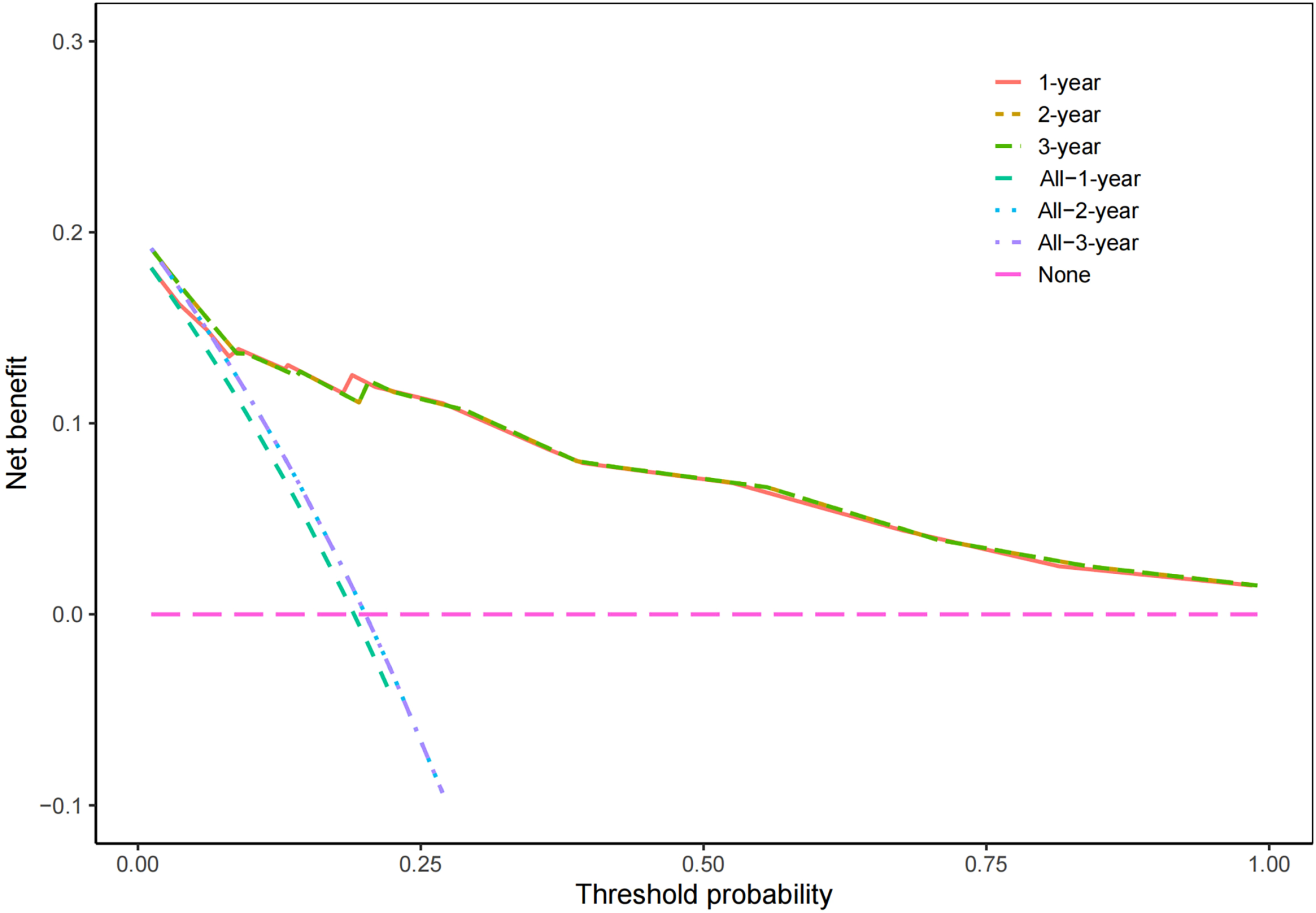
Decision curve analysis for the nomogram.

According to the results of multivariate Cox regression analysis, a nomogram for predicting OS of HB patients receiving preoperative NACT was constructed using SIRI, POSTTEXT staging, MiVI, and AFP (**Figure 7**). Based on the points assigned to the four independent prognostic factors in the nomogram, we can add up the points for each factor to obtain a total point and the corresponding probabilities of OS at 1, 2, and 3 years. A higher total point indicates a lower probability of OS. The C-index of the nomogram was 0.85 (95% CI: 0.78-0.91, p-value = 1.43e-27) and the calibration curves are shown in **Figure 8**, indicating good overall fit and predictive ability. The decision curve analysis (DCA) is presented in **Figure 9**, where the net benefit of the nomogram is greater than 0 over the entire threshold probability, indicating a significant clinical utility of this nomogram.

## 4 Discussion

Many studies have shown that tumor development and progression are closely related to inflammatory response (36). First of all, tumor cells can release a variety of inflammatory factors, such as interleukin (IL)-1, IL-6, IL-8, tumor necrosis factor (TNF) and so on to functionally shape their microenvironment, and these factors can dilate blood vessels and attract innate immune cells and adaptive immune cells to the tumor site, which in turn cause local and systemic inflammatory responses (37). Inflammation, in turn, can promote tumorigenesis and progression, and numerous studies have shown that neutrophils, lymphocytes, monocytes and platelets play an important role in the tumor microenvironment. For example, neutrophils affect tumor development by secreting a variety of factors, such as matrix metalloproteinase-9 (MMP-9), reactive oxygen species (ROS) and reactive nitrogen species (RNS), which promote tumor growth, metastasis, angiogenesis, and immunosuppression (38–40), and monocytes can secrete a variety of growth factors and cytokines, such as IL-6, TNF-α, vascular endothelial growth factor (VEGF) and platelet-derived growth factor (PDGF), which can stimulate tumor cell proliferation and metastasis. In addition, monocytes can mature into tumor-associated macrophages and promote angiogenesis by secreting various growth factors and cytokines (41–44). Platelets can directly interact with tumor cells, promote their proliferation and metastasis, and form a physical barrier around tumor cells, protecting them from immune-mediated lysis by natural killer cells (NK) (45). On the other hand, lymphocytes, especially CD8+ T lymphocytes, play a crucial role in the anti-tumor immune response. CD8+ T lymphocytes mediate cytotoxic responses by releasing inflammatory factors like Interferon-gamma (IFN-γ) and TNF-α, inhibiting tumor growth, proliferation, and metastasis (46). Therefore, under the interaction between cancer cells and inflammatory cells, patients with malignant tumors often exhibit systemic inflammatory responses.

In addition to promoting the occurrence and development of tumors, numerous clinical studies have demonstrated that various inflammatory indexes based on different combinations of inflammatory cells can be used to predict the prognosis of cancer patients. Common indexes such as SIRI, NLR, PLR, and SII can reflect systemic inflammatory responses, and higher levels of these indexes have been associated with worse prognosis in cancer patients (16). Among them, SIRI as a prognostic factor for tumor patients has been extensively studied only in recent years (25, 47–49). It can effectively reflect the inflammatory status of the body as well as the severity of the tumor. Therefore, SIRI has been used to evaluate the prognosis of patients with malignant tumors. For example, a study conducted by Cristina Valero et al. involving 824 patients with head and neck squamous cell carcinoma (HNSCC) found that SIRI is an independent prognostic factor in HNSCC. Patients with higher SIRI experienced a significant decrease in disease-specific survival (50). However, it is worth noting that SIRI is still relatively understudied in the field of pediatric tumors (51). According to available literature searches, there is currently no relevant study on the association between SIRI and pediatric abdominal tumors. Also, there are few studies on the correlation between preoperative inflammatory indexes and the prognosis of hepatoblastoma. In a recent study, Tan Xie analyzed data from 101 HB patients and 101 children with indirect inguinal hernia, and found that both NLR and PLR were prognostic factors for HB. Additionally, NLR was found to be an independent prognostic factor for OS among HB patients (52). However, the study did not differentiate between patients undergoing primary hepatic resection and those receiving preoperative neoadjuvant chemotherapy, which tends to have a large difference in survival (53, 54).

Our study divided HB patients who received preoperative NACT into different groups based on the optimal cutoff values of the inflammation indexes. KM curve of 5-year OS rates in different groups showed that all groups with higher preoperative inflammation indexes had significantly lower survival rates than the low group, and these differences were statistically significant. This suggests that higher preoperative inflammatory indexes are associated with adverse prognosis in HB patients. Furthermore, univariate Cox regression analysis showed that NLR, PLR, SII, and SIRI were all associated with the OS of the patients. However, when considering these inflammatory indexes together in a multivariate Cox regression analysis, only SIRI emerged as an independent prognostic factor, while NLR, PLR, and SII were not. This finding indicates that SIRI has a better predictive value than the other preoperative inflammatory indexes in terms of assessing the OS of HB patients who received preoperative NACT. Further analysis of the relationship between preoperative SIRI and the clinicopathologic characteristics of those patients revealed significant differences in the NLR, SII, POSTTEXT staging among different SIRI groups. A higher preoperative SIRI value indicates a more severe inflammatory state and a higher tumor staging in patients. Moreover, apart from SIRI and POSTTEXT staging, this study identified AFP and MiVI as independent factors influencing the prognosis of pediatric HB patients, which is consistent with previous literature. According to the results of the Children’s Hepatic Tumors International Collaboration (CHIC), a low level of AFP (< 100 ng/ml) was associated with the worst outcome (7, 55). De Ioris, Maretta found that HB patients with low level of AFP were characterized by a high-risk subgroup with extensive disease, poor chemotherapy response and a poor outcome (56). Additionally, MiVI, which may indicate occult micrometastasis of the tumor, was found to be associated with poor prognosis in patients with HB (57). Our study also found no statistically significant difference in MiVI and AFP values between patients in the high SIRI group and those in the low SIRI group. These results suggest that SIRI can serve as a clinical tool to identify patients with a potentially poor prognosis who have high AFP and negative MiVI values.

The treatment decisions for HB primarily rely on preoperative imaging and histopathologic assessment, without considering the potential impact of systemic inflammation on the formulation of appropriate treatment strategies. This is particularly pertinent for children with advanced HB, such as those with POSTTEXT IV and POSTTEXT III tumors with macrovascular involvement, where controversies still exist regarding the most suitable treatment plan. Early studies more recommend total hepatectomy with liver transplantation (58), as this approach has been shown to significantly improve their survival rate (59, 60). However, accumulating evidence from standardized chemotherapy regimens and surgical experience suggests that the prognosis after surgical resection is comparable to that of liver transplantation, while also avoiding long-term complications associated with postoperative immunosuppressive therapy (61–64). On the other hand, studies have demonstrated poor survival outcomes in HB patients undergoing salvage liver transplantation after tumor recurrence (65, 66). These conflicting findings highlight the need for careful consideration by clinicians when deciding between surgical resection and liver transplantation for children with advanced HB. In this context, preoperative SIRI, AFP,and MiVI, in combination with POSTTEXT staging, can offer valuable insights to clinicians, enabling them to comprehensively assess the prognosis of pediatric HB patients from different perspectives. The nomogram prediction model, which incorporates SIRI and clinicopathologic factors, has shown good predictive ability, suggesting its potential usefulness as a valuable tool for clinicians in formulating appropriate personalized treatment strategies. Moreover, alternative techniques, such as transarterial chemoembolization (TACE), radiofrequency ablation (RFA), and high-intensity focused ultrasound (HIFU), have been utilized in pediatric oncology (67–69). These techniques hold promise for patients with high preoperative inflammatory indexes, as they may effectively control tumor growth and allow for postponement of surgical treatment, leading to potentially improved outcomes for these children. However, as there is no relevant literature on this specific aspect, the feasibility and efficacy of such alternative treatments in the context of high preoperative inflammatory indexes would require further investigation through well-designed clinical trials.

Although this study demonstrates the predictive value of preoperative SIRI for the prognosis of HB patients after radical resection, some limitations must be acknowledged. The relatively small sample size and retrospective single-center design may have introduced some confounding factors and biases. To overcome these limitations and provide more definitive evidence, larger, well-designed multicenter prospective studies are warranted. Recently, the COG trial AHEP0731 indicated that patients with unresectable HB had the best (to date) published outcomes with neoadjuvant C5VD (70, 71). Whether our findings are relevant in the C5VD context has not been determined, which minimizes the clinical impact of these findings. Also, the prognostic value of SIRI for those HB patients who have liver transplantation remains to be further investigated. Finally, our study did not demonstrate the prognostic value of preoperative inflammatory indexes for EFS, which is an important factor in the prediction of relapse. We hope that future researchers will identify improved indexes to address this deficiency.

## 5 Conclusion

For pediatric HB patients who undergo preoperative NACT, the preoperative SIRI level can serve as a simple, effective, low-cost, and non-invasive prognostic indicator for predicting OS. Its predictive value is superior to NLR, SII, and PLR. The OS nomogram prediction model constructed based on SIRI, POSTTEXT staging, MiVI, and AFP, can help guide clinicians in formulating personalized treatment plans.

## Data availability statement

The raw data supporting the conclusions of this article will be made available by the authors, without undue reservation.

## Ethics statement

The studies involving humans were approved by Ethics Committee of the Capital Institute of Pediatrics. The studies were conducted in accordance with the local legislation and institutional requirements. Written informed consent for participation in this study was provided by the participants’ legal guardians/next of kin.

## Author contributions

CZ: Data curation, Formal Analysis, Methodology, Writing – original draft. SY: Data curation, Writing – original draft. WL: Data curation, Visualization, Writing – review & editing. MD: Methodology, Project administration, Supervision, Writing – review & editing. LL: Funding acquisition, Investigation, Methodology, Project administration, Resources, Supervision, Writing – review & editing.
